# Differential early subcortical involvement in genetic FTD within the GENFI cohort

**DOI:** 10.1016/j.nicl.2021.102646

**Published:** 2021-03-29

**Authors:** Martina Bocchetta, Emily G. Todd, Georgia Peakman, David M. Cash, Rhian S. Convery, Lucy L. Russell, David L. Thomas, Juan Eugenio Iglesias, John C. van Swieten, Lize C. Jiskoot, Harro Seelaar, Barbara Borroni, Daniela Galimberti, Raquel Sanchez-Valle, Robert Laforce Jr, Fermin Moreno, Matthis Synofzik, Caroline Graff, Mario Masellis, Maria Carmela Tartaglia, James B. Rowe, Rik Vandenberghe, Elizabeth Finger, Fabrizio Tagliavini, Alexandre de Mendonça, Isabel Santana, Chris R. Butler, Simon Ducharme, Alexander Gerhard, Adrian Danek, Johannes Levin, Markus Otto, Sandro Sorbi, Isabelle Le Ber, Florence Pasquier, Jonathan D. Rohrer, on behalf of the Genetic Frontotemporal dementia Initiative (GENFI), Sónia Afonso, Maria Rosario Almeida, Sarah Anderl-Straub, Christin Andersson, Anna Antonell, Silvana Archetti, Andrea Arighi, Mircea Balasa, Myriam Barandiaran, Nuria Bargalló, Robart Bartha, Benjamin Bender, Alberto Benussi, Maxime Bertoux, Anne Bertrand, Valentina Bessi, Sandra Black, Sergi Borrego-Ecija, Jose Bras, Alexis Brice, Rose Bruffaerts, Agnès Camuzat, Marta Cañada, Valentina Cantoni, Paola Caroppo, Miguel Castelo-Branco, Olivier Colliot, Thomas Cope, Vincent Deramecourt, María de Arriba, Giuseppe Di Fede, Alina Díez, Diana Duro, Chiara Fenoglio, Camilla Ferrari, Catarina B. Ferreira, Nick Fox, Morris Freedman, Giorgio Fumagalli, Aurélie Funkiewiez, Alazne Gabilondo, Roberto Gasparotti, Serge Gauthier, Stefano Gazzina, Giorgio Giaccone, Ana Gorostidi, Caroline Greaves, Rita Guerreiro, Carolin Heller, Tobias Hoegen, Begoña Indakoetxea, Vesna Jelic, Hans-Otto Karnath, Ron Keren, Gregory Kuchcinski, Tobias Langheinrich, Thibaud Lebouvier, Maria João Leitão, Albert Lladó, Gemma Lombardi, Sandra Loosli, Carolina Maruta, Simon Mead, Lieke Meeter, Gabriel Miltenberger, Rick van Minkelen, Sara Mitchell, Katrina Moore, Benedetta Nacmias, Annabel Nelson, Jennifer Nicholas, Linn Öijerstedt, Jaume Olives, Sebastien Ourselin, Alessandro Padovani, Jessica Panman, Janne M. Papma, Yolande Pijnenburg, Cristina Polito, Enrico Premi, Sara Prioni, Catharina Prix, Rosa Rademakers, Veronica Redaelli, Daisy Rinaldi, Tim Rittman, Ekaterina Rogaeva, Adeline Rollin, Pedro Rosa-Neto, Giacomina Rossi, Martin Rossor, Beatriz Santiago, Dario Saracino, Sabrina Sayah, Elio Scarpini, Sonja Schönecker, Elisa Semler, Rachelle Shafei, Christen Shoesmith, Imogen Swift, Miguel Tábuas-Pereira, Mikel Tainta, Ricardo Taipa, David Tang-Wai, Paul Thompson, Hakan Thonberg, Carolyn Timberlake, Pietro Tiraboschi, Philip Van Damme, Mathieu Vandenbulcke, Michele Veldsman, Ana Verdelho, Jorge Villanua, Jason Warren, Carlo Wilke, Ione Woollacott, Elisabeth Wlasich, Henrik Zetterberg, Miren Zulaica

**Affiliations:** a Dementia Research Centre, Department of Neurodegenerative Disease, UCL Queen Square Institute of Neurology, University College London, London, United Kingdom; b Centre for Medical Image Computing, Department of Medical Physics and Biomedical Engineering, University College London, London, United Kingdom; c Neuroradiological Academic Unit, UCL Queen Square Institute of Neurology, University College London, London, United Kingdom; d Martinos Center for Biomedical Imaging, Massachusetts General Hospital and Harvard Medical School, USA; e Computer Science and Artificial Intelligence Laboratory, Massachusetts Institute of Technology, USA; f Department of Neurology and Alzheimer Center, Erasmus Medical Center Rotterdam, the Netherlands; g Centre for Neurodegenerative Disorders, Neurology Unit, Department of Clinical and Experimental Sciences, University of Brescia, Brescia, Italy; h Department of Biomedical, Surgical and Dental Sciences, University of Milan, Milan, Italy; i Fondazione IRCCS Ca’ Granda, Ospedale Maggiore Policlinico, Milan, Italy; j Neurology Department, Hospital Clinic, Institut d’Investigacions Biomèdiques, Barcelona, Spain; k Clinique Interdisciplinaire de Mémoire, Département des Sciences Neurologiques, CHU de Québec, Faculté de Médecine, Université Laval, Québec, Canada; l Hospital Universitario Donostia, San Sebastian, Spain; m Department of Cognitive Neurology, Center for Neurology, Hertie-Institute for Clinical Brain Research, Tübingen, Germany; n Karolinska Institutet, Department NVS, Division of Neurogeriatrics, Stockholm, Sweden; o Unit for Hereditray Dementia, Theme Aging, Karolinska University Hospital-Solna Stockholm Sweden; p Campbell Cognitive Neurology Research Unit, Sunnybrook Research Institute, Toronto, ON, Canada; q Toronto Western Hospital, Tanz Centre for Research in Neurodegenerative Disease, Toronto, ON, Canada; r Department of Clinical Neurosciences and Cambridge University Hospitals NHS Trust and Medical Research Council Cognition and Brain Sciences Unit, University of Cambridge, Cambridge, United Kingdom; s Laboratory for Cognitive Neurology, Department of Neurosciences, KU Leuven, Leuven, Belgium; t Department of Clinical Neurological Sciences, University of Western Ontario, London, ON, Canada; u Fondazione Istituto di Ricovero e Cura a Carattere Scientifico, Istituto Neurologico Carlo Besta, Milan, Italy; v Faculty of Medicine, University of Lisbon, Lisbon, Portugal; w Neurology Department, Centro Hospitalar e Universitário de Coimbra, Portugal; x Department of Clinical Neurology, University of Oxford, Oxford, United Kingdom; y Department of Neurology and Neurosurgery, McGill University, Montreal, Quebec, Canada; z Division of Neuroscience and Experimental Psychology, Wolfson Molecular Imaging Centre, University of Manchester, Manchester, United Kingdom; aa Departments of Geriatric Medicine and Nuclear Medicine, University of Duisburg-Essen, Germany; ab Neurologische Klinik und Poliklinik, Ludwig-Maximilians-Universität, Munich German Center for Neurodegenerative Diseases (DZNE), Munich Munich Cluster of Systems Neurology, Munich, Germany; ac Department of Neurology, University Hospital Ulm, Ulm, Germany; ad Department of Neuroscience, Psychology, Drug Research and Child Health, University of Florence, Florence, Italy; ae Sorbonne Université, Paris Brain Institute – Institut du Cerveau– ICM, Inserm U1127, CNRS UMR 7225, AP-HP - Hôpital Pitié-Salpêtrière, Paris, France; af Centre deréférence des démences rares ou précoces, IM2A, Département de Neurologie, AP-HP - Hôpital Pitié-Salpêtrière, Paris, France; ag Département de Neurologie, AP-HP - Hôpital Pitié-Salpêtrière, Paris, France; ah Univ Lille, France; ai Inserm 1172 Lille, France; aj CHU, CNR-MAJ, Labex Distalz, LiCENDLille, France; ak Dementia Research Centre, Department of Neurodegenerative Disease, UCL Institute of Neurology, Queen Square, London, UK; al Baycrest Health Sciences, Rotman Research Institute, University of Toronto, Toronto, Canada; am Fondazione IRCCS Ca’ Granda Ospedale Maggiore Policlinico, Neurodegenerative Diseases Unit, Milan, Italy, University of Milan, Centro Dino Ferrari, Milan, Italy; an Centre de référence des démences rares ou précoces, IM2A, Département de Neurologie, AP-HP - Hôpital Pitié-Salpêtrière, Paris, France, Sorbonne Université, Paris Brain Institute – Institut du Cerveau – ICM, Inserm U1127, CNRS UMR 7225, AP-HP - Hôpital Pitié-Salpêtrière, Paris, France; ao Neuroscience Area, Biodonostia Health Research Insitute, San Sebastian, Gipuzkoa, Spain; ap Neuroradiology Unit, University of Brescia, Brescia, Italy; aq Alzheimer Disease Research Unit, McGill Centre for Studies in Aging, Department of Neurology & Neurosurgery, McGill University, Montreal, Québec, Canada; ar Neurology, ASST Brescia Hospital, Brescia, Italy; as Fondazione IRCCS Istituto Neurologico Carlo Besta, Milano, Italy; at Neuroscience Area, Biodonostia Health Research Insitute, San Sebastian, Gipuzkoa, Spain; au Dementia Research Centre, Department of Neurodegenerative Disease, UCL Institute of Neurology, Queen Square, London, UK; av Center for Neurodegenerative Science, Van Andel Institute, Grand Rapids, Michigan, MI 49503, USA; aw Dementia Research Centre, Department of Neurodegenerative Disease, UCL Institute of Neurology, Queen Square, London, UK; ax Neurologische Klinik, Ludwig-Maximilians-Universität München, Munich, Germany; ay Cognitive Disorders Unit, Department of Neurology, Donostia University Hospital, San Sebastian, Gipuzkoa, Spain, Neuroscience Area, Biodonostia Health Research Insitute, San Sebastian, Gipuzkoa, Spain; az Division of Clinical Geriatrics, Karolinska Institutet, Stockholm, Sweden; ba Division of Neuropsychology, Hertie-Institute for Clinical Brain Research and Center of Neurology, University of Tübingen, Tübingen, Germany; bb The University Health Network, Toronto Rehabilitation Institute, Toronto, Canada; bc Univ Lille, France, Inserm 1172, Lille, France, CHU, CNR-MAJ, Labex Distalz, LiCEND Lille, France; bd Division of Neuroscience and Experimental Psychology, Wolfson Molecular Imaging Centre, University of Manchester, Manchester, UK, Manchester Centre for Clinical Neurosciences, Department of Neurology, Salford Royal NHS Foundation Trust, Manchester, UK; be Univ Lille, France, Inserm 1172, Lille, France, CHU, CNR-MAJ, Labex Distalz, LiCEND Lille, France; bf Centre of Neurosciences and Cell Biology, Universidade de Coimbra, Coimbra, Portugal; bg Alzheimer’s disease and Other Cognitive Disorders Unit, Neurology Service, Hospital Clínic, Barcelona, Spain; bh Department of Neuroscience, Psychology, Drug Research and Child Health, University of Florence, Florence, Italy; bi Neurologische Klinik, Ludwig-Maximilians-Universität München, Munich, Germany; bj Carolina Maruta - Laboratory of Language Research, Centro de Estudos Egas Moniz, Faculty of Medicine, University of Lisbon, Lisbon, Portugal; bk MRC Prion Unit, Department of Neurodegenerative Disease, UCL Institute of Neurology, Queen Square, London, UK; bl Department of Neurology, Erasmus Medical Center, Rotterdam, Netherlands; bm Faculty of Medicine, University of Lisbon, Lisbon, Portugal; bn Department of Clinical Genetics, Erasmus Medical Center, Rotterdam, Netherlands; bo Sunnybrook Health Sciences Centre, Sunnybrook Research Institute, University of Toronto, Toronto, Canada; bp Dementia Research Centre, Department of Neurodegenerative Disease, UCL Institute of Neurology, Queen Square, London UK; bq Department of Neuroscience, Psychology, Drug Research and Child Health, University of Florence, Florence, Italy; br Dementia Research Centre, Department of Neurodegenerative Disease, UCL Institute of Neurology, Queen Square, London, UK; bs Department of Medical Statistics, London School of Hygiene and Tropical Medicine, London, UK; bt Center for Alzheimer Research, Division of Neurogeriatrics, Department of Neurobiology, Care Sciences and Society, Bioclinicum, Karolinska Institutet, Solna, Sweden, Unit for Hereditary Dementias, Theme Aging, Karolinska University Hospital, Solna, Sweden; bu Alzheimer’s disease and Other Cognitive Disorders Unit, Neurology Service, Hospital Clínic, Barcelona, Spain; bv School of Biomedical Engineering & Imaging Sciences, King's College London, London, UK; bw Centre for Neurodegenerative Disorders, Department of Clinical and Experimental Sciences, University of Brescia, Italy; bx Department of Neurology, Erasmus Medical Center, Rotterdam, Netherlands; by Department of Neurology, Erasmus Medical Center, Rotterdam; bz Amsterdam University Medical Centre, Amsterdam VUmc, Amsterdam, Netherlands; ca Department of Biomedical, Experimental and Clinical Sciences “Mario Serio”, Nuclear Medicine Unit, University of Florence, Florence, Italy; cb Stroke Unit, ASST Brescia Hospital, Brescia, Italy; cc Fondazione IRCCS Istituto Neurologico Carlo Besta, Milano, Italy; cd Neurologische Klinik, Ludwig-Maximilians-Universität München, Munich, Germany; ce [as London Ontario geneticist] - Department of Neurosciences, Mayo Clinic, Jacksonville, Florida, USA; cf Fondazione IRCCS Istituto Neurologico Carlo Besta, Milano, Italy; cg Centre de référence des démences rares ou précoces, IM2A, Département de Neurologie, AP-HP - Hôpital Pitié-Salpêtrière, Paris, France, Sorbonne Université, Paris Brain Institute – Institut du Cerveau – ICM, Inserm U1127, CNRS UMR 7225, AP-HP - Hôpital Pitié-Salpêtrière, Paris, France, Département de Neurologie, AP-HP - Hôpital Pitié-Salpêtrière, Paris, France, Reference Network for Rare Neurological Diseases (ERN-RND), France; ch Department of Clinical Neurosciences, University of Cambridge, Cambridge, UK; ci Tanz Centre for Research in Neurodegenerative Diseases, University of Toronto, Toronto, Canada; cj CHU, CNR-MAJ, Labex Distalz, LiCEND Lille, France; ck Translational Neuroimaging Laboratory, McGill Centre for Studies in Aging, McGill University, Montreal, Québec, Canada; cl Fondazione IRCCS Istituto Neurologico Carlo Besta, Milano, Italy; cm Dementia Research Centre, Department of Neurodegenerative Disease, UCL Institute of Neurology, Queen Square, London, UK; cn Neurology Department, Centro Hospitalar e Universitario de Coimbra, Coimbra, Portugal; co Sorbonne Université, Paris Brain Institute – Institut du Cerveau – ICM, Inserm U1127, CNRS UMR 7225, AP-HP - Hôpital Pitié-Salpêtrière, Paris, France, Inria, Aramis project-team, F-75013, Paris, France, Centre de référence des démences rares ou précoces, IM2A, Département de Neurologie, AP-HP - Hôpital Pitié-Salpêtrière, Paris, France; cp Sorbonne Université, Paris Brain Institute – Institut du Cerveau – ICM, Inserm U1127, CNRS UMR 7225, AP-HP - Hôpital Pitié-Salpêtrière, Paris, France; cq Fondazione IRCCS Ca’ Granda Ospedale Maggiore Policlinico, Neurodegenerative Diseases Unit, Milan, Italy, University of Milan, Centro Dino Ferrari, Milan, Italy; cr Neurologische Klinik, Ludwig-Maximilians-Universität München, Munich, Germany; cs Department of Neurology, University of Ulm, Ulm; ct Dementia Research Centre, Department of Neurodegenerative Disease, UCL Institute of Neurology, Queen Square, London, UK; cu Department of Clinical Neurological Sciences, University of Western Ontario, London, Ontario, Canada; cv Department of Neurodegenerative Disease, Dementia Research Centre, UCL Institute of Neurology, Queen Square, London, UK; cw Neurology Department, Centro Hospitalar e Universitario de Coimbra, Coimbra, Portugal; cx Neuroscience Area, Biodonostia Health Research Insitute, San Sebastian, Gipuzkoa, Spain; cy Neuropathology Unit and Department of Neurology, Centro Hospitalar do Porto - Hospital de Santo António, Oporto, Portugal; cz The University Health Network, Krembil Research Institute, Toronto, Canada; da Division of Neuroscience and Experimental Psychology, Wolfson Molecular Imaging Centre, University of Manchester, Manchester, UK; db Center for Alzheimer Research, Division of Neurogeriatrics, Karolinska Institutet, Stockholm, Sweden; dc Department of Clinical Neurosciences, University of Cambridge, Cambridge, UK; dd Fondazione IRCCS Istituto Neurologico Carlo Besta, Milano, Italy; de Neurology Service, University Hospitals Leuven, Belgium, Laboratory for Neurobiology, VIB-KU Leuven Centre for Brain Research, Leuven, Belgium; df Geriatric Psychiatry Service, University Hospitals Leuven, Belgium, Neuropsychiatry, Department of Neurosciences, KU Leuven, Leuven, Belgium; dg Nuffield Department of Clinical Neurosciences, Medical Sciences Division, University of Oxford, Oxford, UK; dh Department of Neurosciences and Mental Health, Centro Hospitalar Lisboa Norte - Hospital de Santa Maria & Faculty of Medicine, University of Lisbon, Lisbon, Portugal; di OSATEK, University of Donostia, San Sebastian, Gipuzkoa, Spain; dj Dementia Research Centre, Department of Neurodegenerative Disease, UCL Institute of Neurology, Queen Square, London, UK; dk Department of Neurodegenerative Diseases, Hertie-Institute for Clinical Brain Research and Center of Neurology, University of Tübingen, Tübingen, Germany, Center for Neurodegenerative Diseases (DZNE), Tübingen, Germany; dl Dementia Research Centre, Department of Neurodegenerative Disease, UCL Institute of Neurology, Queen Square, London, UK; dm Neurologische Klinik, Ludwig-Maximilians-Universität München, Munich, Germany; dn Dementia Research Institute, Department of Neurodegenerative Disease, UCL Institute of Neurology, Queen Square, London, UK; do Neuroscience Area, Biodonostia Health Research Insitute, San Sebastian, Gipuzkoa, Spain; a Instituto Ciencias Nucleares Aplicadas a Saude, Universidade de Coimbra, Coimbra, Portugal; b Faculty of Medicine, University of Coimbra, Coimbra, Portugal; c Department of Neurology, University of Ulm, Ulm, Germany; d Department of Clinical Neuroscience, Karolinska Institutet, Stockholm, Sweden; e Alzheimer’s disease and Other Cognitive Disorders Unit, Neurology Service, Hospital Clínic, Barcelona, Spain; f Biotechnology Laboratory, Department of Diagnostics, ASST Brescia Hospital, Brescia, Italy; g Fondazione IRCCS Ca’ Granda Ospedale Maggiore Policlinico, Neurodegenerative Diseases Unit, Milan, Italy, University of Milan, Centro Dino Ferrari, Milan, Italy; h Alzheimer’s disease and Other Cognitive Disorders Unit, Neurology Service, Hospital Clínic, Barcelona, Spain; i Cognitive Disorders Unit, Department of Neurology, Donostia University Hospital, San Sebastian, Gipuzkoa, Spain, euroscience Area, Biodonostia Health Research Institute, San Sebastian, Gipuzkoa, Spain; j Imaging Diagnostic Center, Hospital Clínic, Barcelona, Spain; k Department of Medical Biophysics, The University of Western Ontario, London, Ontario, Canada, Centre for Functional and Metabolic Mapping, Robarts Research Institute, The University of Western Ontario, London, Ontario, Canada; l Department of Diagnostic and Interventional Neuroradiology, University of Tübingen, Tübingen, Germany; m Centre for Neurodegenerative Disorders, Department of Clinical and Experimental Sciences, University of Brescia, Italy; n Inserm 1172, Lille, France, CHU, CNR-MAJ, Labex Distalz, LiCEND Lille, France; o Sorbonne Université, Paris Brain Institute – Institut du Cerveau – ICM, Inserm U1127, CNRS UMR 7225, AP-HP - Hôpital Pitié-Salpêtrière, Paris, France, Inria, Aramis project-team, F-75013, Paris, France, Centre pour l'Acquisition et le Traitement des Images, Institut du Cerveau et la Moelle, Paris, France; p Department of Neuroscience, Psychology, Drug Research, and Child Health, University of Florence, Florence, Italy; q Sunnybrook Health Sciences Centre, Sunnybrook Research Institute, University of Toronto, Toronto, Canada; r Alzheimer’s disease and Other Cognitive Disorders Unit, Neurology Service, Hospital Clínic, Barcelona, Spain; s Center for Neurodegenerative Science, Van Andel Institute, Grand Rapids, Michigan, MI 49503, USA; t Sorbonne Université, Paris Brain Institute – Institut du Cerveau – ICM, Inserm U1127, CNRS UMR 7225, AP-HP - Hôpital Pitié-Salpêtrière, Paris, France, Reference Network for Rare Neurological Diseases (ERN-RND), France; u Laboratory for Cognitive Neurology, Department of Neurosciences, KU Leuven, Leuven, Belgium; v Sorbonne Université, Paris Brain Institute – Institut du Cerveau – ICM, Inserm U1127, CNRS UMR 7225, AP-HP - Hôpital Pitié-Salpêtrière, Paris, France; w CITA Alzheimer, San Sebastian, Gipuzkoa, Spain; x Centre for Neurodegenerative Disorders, Neurology Unit, Department of Clinical and Experimental Sciences, University of Brescia, Brescia, Italy; y Fondazione IRCCS Istituto Neurologico Carlo Besta, Milano, Italy; z Faculty of Medicine, University of Coimbra, Coimbra, Portugal; aa Sorbonne Université, Paris Brain Institute – Institut du Cerveau – ICM, Inserm U1127, CNRS UMR 7225, AP-HP - Hôpital Pitié-Salpêtrière, Paris, France, Inria, Aramis project-team, F-75013, Paris, France, Centre pour l'Acquisition et le Traitement des Images, Institut du Cerveau et la Moelle, Paris, France; ab Department of Clinical Neuroscience, University of Cambridge, Cambridge, UK; ac Univ Lille, France, Inserm 1172, Lille, France, CHU, CNR-MAJ, Labex Distalz, LiCEND Lille, France; ad Neuroscience Area, Biodonostia Health Research Insitute, San Sebastian, Gipuzkoa, Spain; ae Fondazione IRCCS Istituto Neurologico Carlo Besta, Milano, Italy; af Neuroscience Area, Biodonostia Health Research Insitute, San Sebastian, Gipuzkoa, Spain; ag Faculty of Medicine, University of Coimbra, Coimbra, Portugal; ah Fondazione IRCCS Ca’ Granda Ospedale Maggiore Policlinico, Neurodegenerative Diseases Unit, Milan, Italy, University of Milan, Centro Dino Ferrari, Milan, Italy; ai Department of Neuroscience, Psychology, Drug Research, and Child Health, University of Florence, Florence, Italy; aj Laboratory of Neurosciences, Institute of Molecular Medicine, Faculty of Medicine, University of Lisbon, Lisbon, Portugal

**Keywords:** Genetic frontotemporal dementia, MRI imaging, Brain volumetry, Presymptomatic stage

## Abstract

**Background:**

Studies have previously shown evidence for presymptomatic cortical atrophy in genetic FTD. Whilst initial investigations have also identified early deep grey matter volume loss, little is known about the extent of subcortical involvement, particularly within subregions, and how this differs between genetic groups.

**Methods:**

480 mutation carriers from the Genetic FTD Initiative (GENFI) were included (198 *GRN*, 202 *C9orf72*, 80 *MAPT*), together with 298 non-carrier cognitively normal controls. Cortical and subcortical volumes of interest were generated using automated parcellation methods on volumetric 3 T T1-weighted MRI scans. Mutation carriers were divided into three disease stages based on their global CDR® plus NACC FTLD score: asymptomatic (0), possibly or mildly symptomatic (0.5) and fully symptomatic (1 or more).

**Results:**

In all three groups, subcortical involvement was seen at the CDR 0.5 stage prior to phenoconversion, whereas in the *C9orf72* and *MAPT* mutation carriers there was also involvement at the CDR 0 stage. In the *C9orf72* expansion carriers the earliest volume changes were in thalamic subnuclei (particularly pulvinar and lateral geniculate, 9–10%) cerebellum (lobules VIIa-Crus II and VIIIb, 2–3%), hippocampus (particularly presubiculum and CA1, 2–3%), amygdala (all subregions, 2–6%) and hypothalamus (superior tuberal region, 1%). In *MAPT* mutation carriers changes were seen at CDR 0 in the hippocampus (subiculum, presubiculum and tail, 3–4%) and amygdala (accessory basal and superficial nuclei, 2–4%). *GRN* mutation carriers showed subcortical differences at CDR 0.5 in the presubiculum of the hippocampus (8%).

**Conclusions:**

*C9orf72* expansion carriers show the earliest and most widespread changes including the thalamus, basal ganglia and medial temporal lobe. By investigating individual subregions, changes can also be seen at CDR 0 in *MAPT* mutation carriers within the limbic system. Our results suggest that subcortical brain volumes may be used as markers of neurodegeneration even prior to the onset of prodromal symptoms.

## Highlights

• Progressive and differential atrophy patterns are seen at presymptomatic stages across genetic groups.
• Very early presymptomatic brain changes are detectable only by looking at small regions.
• *C9orf72* expansion carriers show the earliest and most widespread changes (cortex, pulvinar, cerebellum).
• *MAPT* mutation carriers show early differences in the dorsolateral temporal cortex, amygdala, and hippocampus.
• Late presymptomatic changes occur in *GRN* mutation carriers in dorsolateral prefrontal cortex, insula, and presubiculum.

## 1 Introduction

Frontotemporal dementia (FTD) is a common cause of early onset dementia. In about a third of the cases it is associated with an autosomal dominant inherited mutation in one of three genes: microtubule-associated protein tau (*MAPT*), progranulin (*GRN*), and chromosome 9 open reading frame 72 (*C9orf72*) (Warren et al., 2013). For each of these genetic groups, there is evidence of a differential pattern of cortical atrophy (Chen and Kantarci, 2020), with changes occurring presymptomatically, up to twenty years before estimated phenoconversion (Rohrer et al., 2015, Cash et al., 2018). Whilst these studies have been highly informative in describing the presence of brain changes in presymptomatic stages of the disease, they have focused less on subcortical structures, and in particular, they have not investigated specific subregions within the deep grey matter. However, due to advanced imaging methods, it is now possible to measure these individual nuclei and subregions *in vivo* on structural magnetic resonance scans, with prior studies in small cohorts showing changes at the symptomatic stage of genetic FTD (Bocchetta et al., 2016, Bocchetta et al., 2018, Bocchetta et al., 2019, Bocchetta et al., 2020), but without any previous investigation of the presymptomatic period. Using data from the Genetic FTD Initiative (GENFI) cohort, we therefore aimed to examine the specific pattern of subcortical changes (including specific subregions), to determine which areas were impaired across the different disease stages of genetic FTD.

## 2 Methods

At the time of the fifth data freeze in the GENFI 2 study (03/03/2015–31/05/2019), 850 participants had been recruited across 24 centres in the United Kingdom, Canada, Italy, the Netherlands, Sweden, Portugal, Germany, France, Spain, and Belgium, of whom 804 had a volumetric T1-weighted magnetic resonance image acquired on a 3 T scanner. Another 26 participants were excluded as the scans were of unsuitable quality due to motion or other imaging artefacts, pathology unlikely to be attributed to FTD, or as they were carriers of mutations in one of the rarer genetic causes of FTD. All the remaining 778 participants were known to be either a carrier of a pathogenic expansion in *C9orf72* or of a pathogenic mutation in *GRN* or *MAPT* (n = 480), or were non-carrier first-degree relatives (n = 298), who therefore acted as controls within the study. All aspects of the study were approved by the local ethics committee for each of the GENFI sites, and written informed consent was obtained from all participants.

All participants underwent a standardized clinical assessment as described previously (Rohrer et al., 2015). This included the CDR® plus NACC FTLD (Miyagawa et al., 2020) which was used to group the mutation carriers into stages: those with a global score of 0 were considered as asymptomatic, those with a score of 0.5 considered as possibly or mildly symptomatic (i.e. prodromal), and those with a score ≥ 1 were considered as fully symptomatic or phenoconverted (Table 1).

**Table 1:** Demographic and clinical characteristic of the cohort divided by genetic group and CDR®+NACC FTLD global scores. Abbreviations: N/A not applicable, FTD frontotemporal dementia, bvFTD behavioural variant FTD, PPA primary progressive aphasia, NOS not otherwise specified, CBS corticobasal syndrome, PSP progressive supranuclear palsy, AD Alzheimer’s disease, ALS amyotrophic lateral sclerosis.

	Non-carriers	*C9orf72* expansion carriers			*MAPT* mutation carriers			*GRN* mutation carriers		
CDR®+NACC FTLD global score		0	0.5	≥1	0	0.5	≥1	0	0.5	≥1
N	298	107	32	63	47	13	20	125	30	43
Age, year	45.8 (12.5)	43.9 (11.7)	49.4 (11.7)	62.9 (9.2)	39.3 (10.6)	47.0 (12.0)	58.2 (10.1)	45.5 (12.0)	52.0 (13.3)	63.6 (8.2)
Sex, male (%)	125 (41.9%)	44 (41.1%)	12 (37.5%)	41 (65.1%)	20 (42.6%)	4 (30.8%)	13 (65.0%)	43 (34.4%)	15 (50%)	21 (48.8%)
Scanners [Siemens Trio/Siemens Skyra/Siemens Prisma/Philips Achieva/GE Discovery MR750]	59/64/79/94/2	32/14/19/42/0	4/4/10/14/0	8/11/27/16/1	11/11/8/16/1	1/1/8/3/0	6/2/10/2/0	39/20/16/45/5	5/7/9/8/1	11/11/14/7/0
Clinical phenotype	N/A	N/A	N/A	49 bvFTD, 6 FTD-ALS, 2 ALS, 2 PPA, 1 PSP, 2 Dementia-NOS, 1 Other	N/A	N/A	16 bvFTD, 1 PPA, 2 Dementia-NOS, 1 PSP	N/A	N/A	24 bvFTD, 17 PPA, 1 CBS, 1 AD

**Fig. 1:**
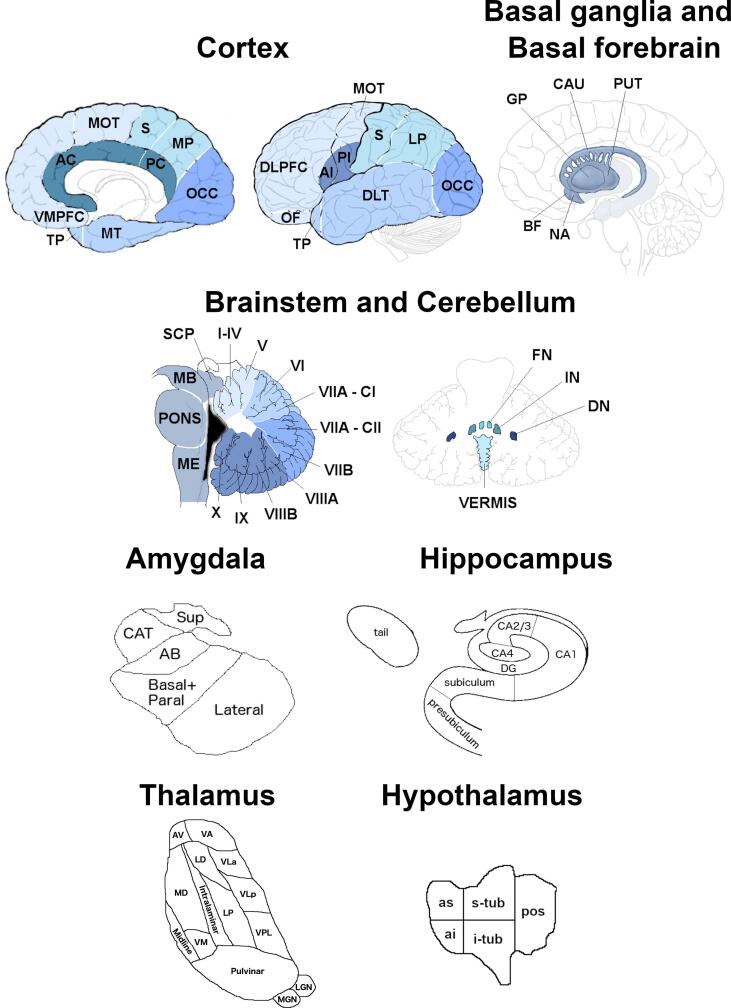
Regions of interest used in the analysis. Abbreviations. Cortical: VMPFC ventromedial prefrontal, TP temporal pole, MT medial temporal, AC anterior cingulate, PC posterior cingulate, MOT motor, S sensory, MP medial parietal, OCC occipital, DLPFC dorsolateral prefrontal, OF orbitofrontal, AI anterior insular, PI posterior insular, DLT dorsolateral temporal, LP lateral parietal; Basal ganglia and Basal forebrain: GP pallidum, CAU caudate, PUT putamen, BF basal forebrain, NA nucleus accumbens; Brainstem: SCP superior cerebellar peduncle, MB midbrain, ME medulla; Cerebellum: VIIA – CI lobule VIIA – Crus I, VIIA – CII lobule VIIA – Crus II, FN fastigial nucleus, IN interposed nucleus, DN dentate nucleus; Amygdala: CAT cortico-amygdaloid transition area, Sup superficial nuclei, AB accessory basal nucleus; Hippocampus: DG dentate gyrus, CA cornu ammonis; Thalamus: AV anteroventral, VA ventral anterior, LD laterodorsal, VLa ventral lateral anterior, MD mediodorsal, LP lateral posterior, VLp ventral lateral posterior, VPL ventral posterolateral, VM ventromedial, LGN lateral geniculate nucleus, MGN medial geniculate nucleus; Hypothalamus: as anterior superior, ai anterior inferior, s-tub superior tuberal, i-tub inferior tuberal, pos posterior.

Participants underwent a 1.1-mm isotropic resolution volumetric T1-weighted magnetic resonance imaging (MRI) on a 3 T scanner (Siemens Trio, Siemens Skyra, Siemens Prisma, Philips Achieva, GE Discovery MR750). Volumetric MRI scans were first bias field corrected and whole brain parcellated using the geodesic information flow (GIF) algorithm (Cardoso et al., 2015), which is based on atlas propagation and label fusion. We combined regions of interest to calculate grey matter volumes of the cortex for 15 regions: orbitofrontal, dorsolateral (DLPFC) and ventromedial prefrontal, motor, anterior and posterior insula, temporal pole, dorsolateral and medial temporal, anterior and posterior cingulate, sensory, medial and lateral parietal, and occipital cortex. Using GIF and customised versions of specific Freesurfer modules (Iglesias et al., 2015, Iglesias et al., 2015, Iglesias et al., 2018, Saygin et al., 2017) that accept the GIF parcellation as inputs (Bocchetta et al., 2020a, Bocchetta et al., 2018, Bocchetta et al., 2019, Bocchetta et al., 2020) we also calculated individual volumes for the following subcortical regions (Fig. 1): i) basal ganglia (nucleus accumbens, caudate, putamen, and globus pallidus, ii) basal forebrain, iii) amygdala (5 regions: lateral nucleus, basal and paralaminar nucleus, accessory basal nucleus, cortico-amygdaloid transition area and the superficial nuclei), iv) hippocampus (7 regions: cornu ammonis CA1, CA2/CA3, CA4, dentate gyrus, subiculum, presubiculum, tail), v) thalamus (14 regions: anteroventral, laterodorsal (LD), lateral posterior, ventral anterior, ventral lateral anterior, ventral lateral posterior, ventral posterolateral, ventromedial, intralaminar, midline, mediodorsal (MD), lateral geniculate (LGN), medial geniculate (MGN) and pulvinar). Volumes for the hypothalamus (5 regions: anterior superior, anterior inferior, superior tuberal (s-tub), inferior tuberal (i-tub), posterior) were computed using the deep convolutional neural network method described in (Billot et al., 2020). We also parcellated the cerebellum (separated into 14 regions: lobules I-IV, V, VI, VIIa-Crus I, VIIa-Crus II, VIIb, VIIIa, VIIIb, IX, X, vermis, dentate nucleus, interposed nucleus and fastigial nucleus (Diedrichsen et al., 2009, Diedrichsen et al., 2011), and brainstem (superior cerebellar peduncle, medulla, pons, and midbrain).

Left and right volumes were summed, and total intracranial volume was computed with SPM12 v6470 (Statistical Parametric Mapping, Wellcome Trust Centre for Neuroimaging, London, UK) running under Matlab R2014b (Math Works, Natick, MA, USA) (Malone et al., 2015). All segmentations were visually checked for quality with only one subject excluded from the cerebellar analyses due to the presence of an arachnoid cyst. Statistical analyses were performed in SPSS software (SPSS Inc., Chicago, IL, USA) version 26, with a linear regression analysis within each genetic group adjusting for age, sex, and scanner type (as there were significant differences between groups for each of these, Table 1), as well as total intracranial volume, with correction for multiple comparisons using the Benjamini & Hochberg method (Benjamini and Hochberg, 1995) using p = 0.05 for false discovery rate. The correction was performed separately for the genetic groups (*MAPT, GRN*, *C9orf72*), while considering the number of comparisons within each of the main regions (cortical, cerebellum, brainstem, thalamus, hypothalamus, amygdala, hippocampus, and other subcortical structures).

## 3 Results

### 3.1 Total brain and cortical volumes

The total brain volume was significantly smaller in all genetic groups with CDR ≥ 1 when compared to controls (8–10% volumetric difference, p < 0.0005). However, it was also significantly smaller in *C9orf72* expansion carriers at CDR 0 and 0.5 (1–3%, p ≤ 0.004) (Supplementary Table 1, Supplementary Fig. 1).

*C9orf72* expansion carriers with a CDR ≥ 1 showed significantly smaller volumes than controls in all cortical regions, with the largest differences in the anterior and posterior insula (24%) (Supplementary Table 1, Supplementary Fig. 2). These two regions, together with the DLPFC, motor, dorsolateral temporal, lateral parietal and occipital cortex, were also significantly smaller in the *C9orf72* expansion carriers with CDR 0 and 0.5 (2–7%, p ≤ 0.006). The temporal pole was significantly smaller than controls in those scoring 0.5 (6%, p = 0.004), while the orbitofrontal, posterior cingulate, sensory and medial parietal cortex were significantly smaller in those scoring 0 (1–4%, p ≤ 0.028), but these differences did not reach statistical significance in those scoring 0.5 (Supplementary Table 1, Supplementary Fig. 2).

*MAPT* mutation carriers with CDR ≥ 1 showed smaller volumes than controls in the temporal regions (32% in the temporal pole), insula (29–30%) and anterior cingulate (12%) (p < 0.0005) (Supplementary Table 1, Supplementary Fig. 2). The dorsolateral temporal cortex was smaller in the CDR 0.5 group (17%, p = 0.003). No difference was found in the CDR 0 group.

*GRN* mutation carriers with CDR ≥ 1 showed smaller volumes in all regions (6–26%, p ≤ 0.012) except the sensory cortex, with the anterior insula being the region with smallest volume (26%, p < 0.0005). *GRN* mutation carriers with CDR 0.5 also showed smaller DLPFC and anterior insula volumes than controls (5–6%, p ≤ 0.013) (Supplementary Table 1, Supplementary Fig. 2). No difference was found in the CDR 0 group.

### 3.2 Basal ganglia

**Fig. 2:**
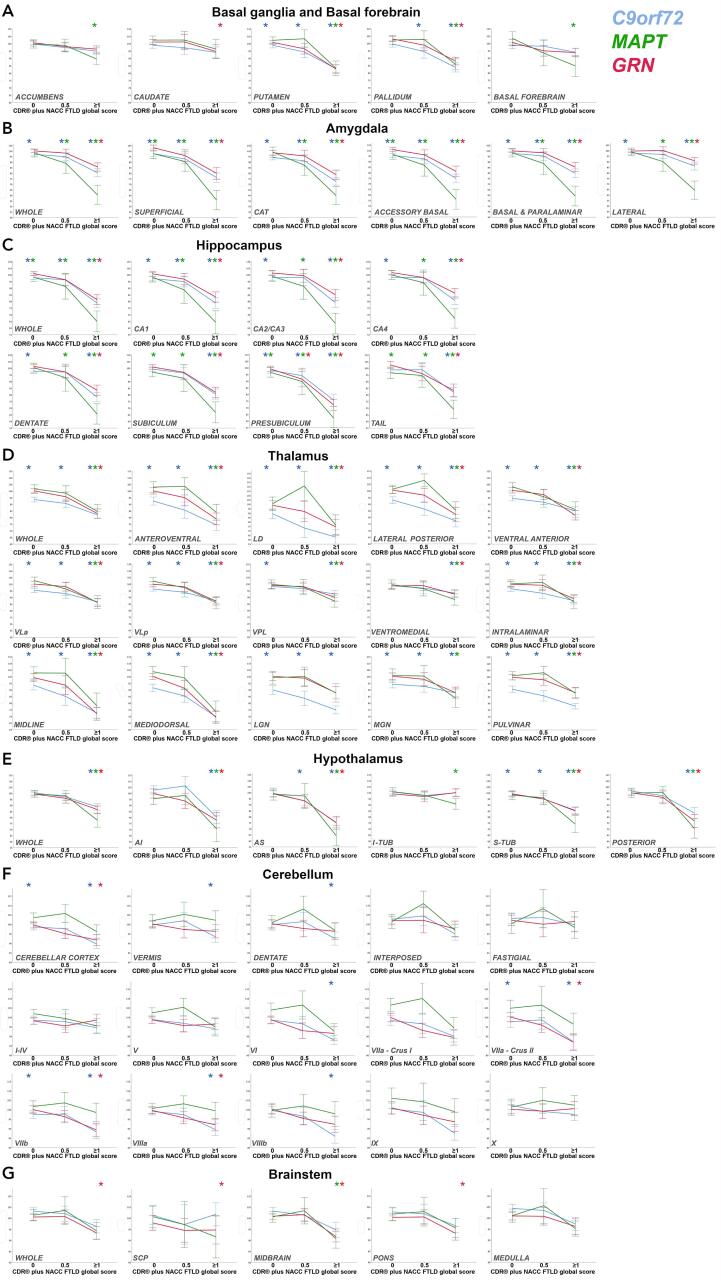
A–G. Plots representing the means and standard error bars for regional brain volumes for each of the stages in *C9orf72*, *MAPT* and *GRN* mutation carriers. Volumes as expressed as % the mean volumes in controls (y axis). * indicates a significant difference from controls after correcting for multiple comparisons. Abbreviations: Amygdala: CAT cortico-amygdaloid transition area; Thalamus: LD laterodorsal, VLa ventral lateral anterior, VLp ventral lateral posterior, VPL ventral posterolateral, LGN lateral geniculate nucleus, MGN medial geniculate nucleus; Hypothalamus: AI anterior inferior, AS anterior superior, I-TUB inferior tuberal, S-TUB superior tuberal; Brainstem: SCP superior cerebellar peduncle.

Among the basal ganglia, the putamen was significantly smaller across all *C9orf72* stages (1–17%, p ≤ 0.0006) (Supplementary Table 1, Fig. 2A), and in the *MAPT* and *GRN* mutation carriers with CDR ≥ 1 (17%, p < 0.0005). *C9orf72* expansion carriers with CDR 0.5 and ≥ 1 showed smaller globus pallidus (6–16%, p ≤ 0.003). *MAPT* mutation carriers with CDR ≥ 1 showed smaller volumes than controls in the nucleus accumbens (11%), and globus pallidus (14%), whilst *GRN* mutation carriers scoring ≥ 1 showed smaller caudate (5%, p = 0.011) and globus pallidus (12%, p < 0.0005). No differences were found for the *MAPT* and *GRN* mutation carriers in the CDR 0 or 0.5 groups.

### 3.3 Basal forebrain

Changes in the basal forebrain were only seen in *MAPT* mutation carriers (Supplementary Table 1, Fig. 2A), and only at the CDR ≥ 1 stage (15% smaller than controls, p < 0.0005).

### 3.4 Amygdala

All amygdalar regions were significantly smaller than controls for all mutation carriers with CDR ≥ 1 (p < 0.0005), with the *MAPT* group showing the largest differences, particularly in the superficial and accessory basal regions (44%) as well as the lateral regions (36%) (Supplementary Table 1, Figure 2B). All regions were significantly smaller in *C9orf72* expansion carriers at CDR 0, and in *MAPT* mutation carriers with CDR 0.5, with smaller volumes at CDR 0 in the superficial and accessory basal nuclei (2–4%, p ≤ 0.034) (Supplementary Table 1, Figure 2B). *GRN* mutation carriers with CDR 0 or 0.5 did not show any significant differences from controls.

### 3.5 Hippocampus

All hippocampal regions were significantly smaller than controls for all mutation carriers with CDR ≥ 1 (p < 0.0005), with *MAPT* mutation carriers being the genetic group with the largest differences (all above 30%) (Supplementary Table 1, Figure 2C). Differences were also seen in all regions in *MAPT* mutation carriers with CDR 0.5 (6–11%, p ≤ 0.019), and in the subiculum, presubiculum and tail (3–4%, p ≤ 0.020) in *MAPT* mutation carriers with CDR 0. In *C9orf72* expansion carriers with CDR 0 there were significantly smaller volumes than controls in all regions except the tail and the subiculum (2–3%, p ≤ 0.015). The presubiculum was the only region significantly smaller in *GRN* mutation carriers with CDR 0.5 (8%, p = 0.016) with no significant differences at CDR 0.

### 3.6 Thalamus

*C9orf72* expansion carriers showed significantly smaller thalamic regions in all stages, with the only exception being the ventromedial nucleus (which only became significant at CDR stage ≥ 1) and the ventral posterolateral nucleus, which did not quite reach statistical significance at CDR 0.5. The most affected regions at CDR 0 were the LD (13%), LGN (10%) and pulvinar (9%) (p < 0.0005) (Supplementary Table 1, Figure 2D). *MAPT* mutation carriers with CDR ≥ 1 showed significantly smaller volumes in all regions except LGN, with the main differences located in the MD, midline and LD regions (22–26%, p < 0.0005) but no differences at earlier stages. *GRN* mutation carriers also only showed significantly smaller regions at CDR ≥ 1, with the main differences located in the MD and midline regions (31%, p < 0.0005), followed by the anteroventral and LD (21–25%, p < 0.0005). No differences were found in the LGN and MGN.

### 3.7 Hypothalamus

All regions were significantly smaller than controls for all mutation carriers with CDR ≥ 1 (p < 0.0005), except for the i-tub regions for *C9orf72* and *GRN* mutation carriers. In the CDR ≥ 1 group *MAPT* mutation carriers had the smallest volumes, with differences above 29% in the posterior and anterior regions (Supplementary Table 1, Figure 2E). *C9orf72* expansion carriers were the only ones showing early differences, with the CDR 0.5 group showing smaller volumes than controls in the anterior superior and s-tub regions (5–7%, p ≤ 0.017), and the CDR 0 group showing smaller volumes than controls in the s-tub region (1%, p = 0.008).

### 3.8 Cerebellum

*C9orf72* and *GRN* mutation carriers with CDR ≥ 1 had smaller volumes than controls in the lobules VIIa-Crus II (13%), VIIb (11–12%) and VIIIa (8–10%) (p ≤ 0.001), with *C9orf72* expansion carriers also showing significant differences in lobules VI (12%), VIIIb (14%), vermis (7%) and the dentate nucleus (7%) (p ≤ 0.007). In addition, the *C9orf72* expansion carriers were the only group with significantly smaller volumes at CDR 0 (lobules VIIa-Crus II and VIIb, 2–3% p ≤ 0.011) (Supplementary Table 1, Figure 2F). No significant difference was found in the *MAPT* group.

### 3.9 Brainstem

*GRN* mutation carriers with CDR ≥ 1 showed smaller volumes in the superior cerebellar peduncle (5%, p = 0.011), midbrain and pons (7–8%, p < 0.0005), while *MAPT* mutation carriers scoring ≥ 1 showed smaller volumes in the midbrain (9%, p < 0.0005) (Supplementary Table 1, Figure 2G). No difference was detected in those with CDR 0 or 0.5, or in *C9orf72* expansion carriers at any stage.

**Fig. 3:**
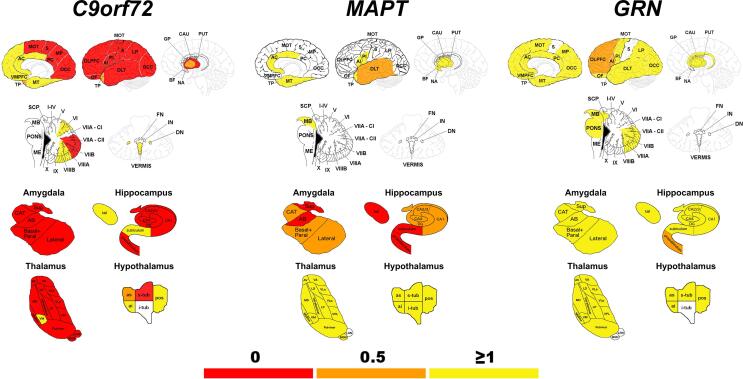
Sequential pattern of neuroanatomical involvement in *C9orf72*, *MAPT* and *GRN*. The colour map indicates the stage defined by CDR®+NACC FTLD global scores when the specific region of interest becomes involved, as significantly smaller than controls.

Fig. 3 summarizes the sequential pattern of neuroanatomical involvement for each of the genetic groups, by indicating at which stage each region resulted significantly smaller than controls.

## 4 Discussion

In this study we have defined the pattern of involvement in subcortical brain regions and specific nuclei in genetic frontotemporal dementia. We have identified gene-specific changes in asymptomatic and prodromal stages through to fully symptomatic stages in *C9orf72*, *MAPT* and *GRN* mutation carriers. By looking at specific regions, in a large cohort of mutation carriers, we were able to identify small changes that occurs very early on in all genetic groups, which might go undetected when looking at the whole brain or at large regions.

The first brain regions showing differences from controls in *C9orf72* expansion carriers without any detectable clinical symptoms were the thalamic regions (the pulvinar, LD and LGN in particular), the putamen, the CA regions with the dentate gyrus and the presubiculum, all the amygdalar regions, the s-tub region in the hypothalamus, the lobule VIIa-Crus II and VIIb of the cerebellum as well as several cortical regions. By the time *C9orf72* expansion carriers reach the symptomatic phase, nearly all the regions in the brain become affected, with the exception of the caudate, nucleus accumbens, basal forebrain, brainstem, and the anterior and inferior cerebellum and the i-tub region of the hypothalamus. These results are in line with previous studies showing widespread involvement of the brain in *C9orf72*-associated FTD, well beyond the classical frontal and temporal regions of FTD (Rohrer et al., 2015, Cash et al., 2018, Bertrand et al., 2018, Lee et al., 2017).

Among the thalamic regions, the pulvinar and LGN were particularly affected in *C9orf72* expansion carriers, which is in line with previous research in both symptomatic and presymptomatic carriers (Bocchetta et al., 2020, Lee et al., 2017) and with the pathological accumulation of TDP-43 and dipeptide repeat proteins in those regions (Vatsavayai et al., 2016, Yang et al., 2017) Atrophy in these regions is linked to hallucinations and other psychotic symptoms as well as the altered processing of pain, features seen more commonly in *C9orf72* expansion carriers than in other forms of FTD (Convery et al., 2020, Ducharme et al., 2017, Fletcher et al., 2015, Kertesz et al., 2013).

Interestingly, the regions affected early in the cerebellum (lobule VIIa-Crus II and VIIb) are connected via the dentate nuclei to the ventral anterior and ventrolateral nuclei of the thalamus and from here to the DLPFC to regulate cognitive functions, and in particular goal-directed complex behaviours (Makris et al., 2003, D'Angelo and Casali, 2013, Palesi et al., 2015). The cerebellum is also connected via the anterior and ventral lateral posterior thalamic regions to the basal ganglia and the parietal and motor cortex (Palesi et al., 2015); all regions affected early in *C9orf72*-associated FTD. Dipeptide repeat proteins are also typical and abundant in the cerebellar cortex (Mackenzie and Neumann, 2016).

The most affected regions within the hippocampus and amygdala are among the ones previously shown to be atrophic in symptomatic *C9orf72* expansion carriers (Bocchetta et al., 2018, Bocchetta et al., 2019) and connected to the temporal and posterior cortex (de Flores et al., 2017). The CA regions are abundant of dipeptide repeat proteins, with or without TDP-43 deposition (Mann and Snowden, 2017).

*C9orf72* expansion carriers at CDR 0 showed reduced volumes in the s-tub region of the hypothalamus and later on in the anterior and posterior hypothalamus, leaving the i-tub as the only spared region as previously reported (Bocchetta et al., 2015). The s-tub region includes the dorso-medial nucleus and lateral hypothalamic area, which regulate appetite and contain neuropeptide-expressing neurons and neuropeptide receptors (Parker and Bloom, 2012). Similarly, volume loss in the posterior hypothalamus and the presence of TDP-43 pathology have been linked to the development of abnormal eating behaviours, typical symptoms of bvFTD (Bocchetta et al., 2015, Piguet et al., 2011).

In *MAPT* mutation carriers, the only regions affected at CDR 0 were the superficial and accessory basal regions of the amygdala, and the subiculum, presubiculum and hippocampal tail. Such early differences in the amygdala could not be detected when looking at its volume as a whole, which only became significantly affected at a later stage. *MAPT* mutation carriers at CDR 0.5 additionally showed smaller volumes in the dorsolateral temporal cortex and in all the other hippocampal and amygdalar regions. Overall, the more medial regions of the amygdala (particularly the superficial, accessory basal and basal and paralaminar) tend to be affected more than the lateral regions. They are connected to key limbic regions and likely related to the development of symptoms associated with abnormal reward and emotional processing. These results are in line with previous *in vivo* studies on symptomatic mutation carriers (Bocchetta et al., 2018, Bocchetta et al., 2019) and with pathological studies: tau deposition is extensively found in the hippocampus and other limbic structures in *MAPT* mutation carriers (Ghetti et al., 2015).

By the time *MAPT* mutation carriers are fully symptomatic, we find lower volumes in the other key regions of the limbic system, such as the insula, anterior cingulate, mediotemporal cortex, nucleus accumbens and basal forebrain. This latter structure, the basal forebrain, was only affected in the *MAPT* genetic group, as previously reported (Convery et al., 2020). Other regions affected in this group include the midbrain, which forms part of a network that regulates emotion perception with the thalamus and amygdala (Liddell et al., 2005). All regions in the hypothalamus were also affected, although mainly in the superior and posterior regions as previously reported in a smaller cohort (Bocchetta et al., 2015). Interestingly, the posterior region includes the mammillary bodies, connected via the fornix to the amygdala and hippocampus. Among the thalamic regions, the MD was the most affected, as previously reported (Bocchetta et al., 2020): this region is connected to brain regions within the limbic network and plays a role in emotional and behavioural regulation, as well as executive function. This sequence of regional involvement and the localisation in the temporal lobe is in line with what has been reported in other studies in *MAPT* mutation carriers (Rohrer et al., 2015, Cash et al., 2018, Whitwell et al., 2012, Olney et al., 2020). The vermis of the cerebellum, another important part of the limbic system, was not affected, in contrast with what was found by another study (Bocchetta et al., 2016). This could be due to the different way of classifying the symptomatic mutation carriers, and the presence of different scanner types and sample characteristics. However, the fact that the cerebellum was overall not affected in *MAPT*-associated FTD is in line with other studies (Rohrer et al., 2015).

*GRN* mutation carriers only showed significant atrophy at the CDR 0.5 stage – this was mainly cortical, affecting the DLPFC, and anterior insula, but there was also subcortical involvement of the presubiculum, a hippocampal region connected to the basal ganglia, frontal and parietal cortex, areas which are typically atrophic in *GRN*, as found here at the symptomatic stages and in other studies (Rohrer et al., 2015, Cash et al., 2018, Olney et al., 2020); and which typically show TDP-43 accumulation (Mann and Snowden, 2017).

Previously described as spared in *GRN* mutation carriers (Bocchetta et al., 2016); we found here that lobule VIIa-Crus II, VIIb and VIIIa are affected later in the disease. These regions are connected, via the thalamus, to the DLPFC and primary sensorimotor cortex (Makris et al., 2003). Within the brainstem, the midbrain, pons, and superior cerebellar peduncle were atrophic, as previously found (Rohrer et al., 2010). The role of the brainstem in FTD is not yet fully understood, but TDP-43 pathology has been found previously in several nuclei of the midbrain and pons (Grinberg et al., 2011). When symptoms were clearly present in *GRN* mutation carriers, all the hypothalamic regions were also smaller than in controls, with the exception of the i-tub (similarly to the *C9orf72* group). This region includes the arcuate nucleus, an important target for metabolic and hormonal signals (Parker and Bloom, 2012). Interestingly, in a previous histological study (and consistent with our findings), TDP-43 inclusions were not found in this region, but were abundant in the anterior, superior and posterior region of the hypothalamus (Cykowski et al., 2016).

*C9orf72* expansion carriers showed by far the earliest and most widespread changes in the brain, compared to *MAPT* and *GRN* mutation carriers. Even the total brain volume was lower in *C9orf72* expansion carriers at CDR 0 whilst only being affected at the fully symptomatic stage in *MAPT* and *GRN* mutation carriers. This result was found previously (Rohrer et al., 2015) and could suggest that *C9orf72*-associated FTD might be associated with a long and slow process of neurodegeneration which could start many decades before the onset of clinical symptoms, as also suggested by Staffaroni *et al* (Staffaroni et al., 2020). *GRN* mutation carriers instead might have a more rapid process which occurs later and closer to symptom onset (Jiskoot et al., 2019). Longitudinal studies, such as in Staffaroni *et al* (Staffaroni et al., 2020) and Whitwell *et al* (Whitwell et al., 2015), looking at the atrophy rates in the different disease stages could potentially provide a definite answer to whether this is the case.

This study has some limitations. Some of the nuclei are very small and we grouped them into combined regions, or clusters of nuclei. In the future, this could be addressed by imaging at higher field strengths (e.g. 7 T), enabling higher spatial resolution. There were differences in age, sex and scanner type for some of the groups, which we have taken into account by including these variables as covariates, although this cannot completely exclude their impact. The CDR 0.5 is smaller than the other groups, and is likely to be heterogeneous including both people who are truly in a mild prodromal stage, and others that score 0.5 due to ‘questionable’ symptoms that might instead be related to affective symptoms during the at-risk period.

By looking at *in vivo* regional volumetry, we have shown here a differential pattern of subcortical changes across severity stages in *C9orf72*, *MAPT* and *GRN* mutation carriers. By looking at a wide range of specific brain regions, for the first time we were able to measure small changes that occur in localized regions in the early stages of genetic FTD. These results suggest that these changes may be used as markers of neurodegeneration in future trials even during preclinical and prodromal periods. Further longitudinal studies, including multimodal imaging looking at brain connectivity networks and including correlations with cognitive and other biomarkers, will be vital to investigate these results further.

## Declaration of Competing Interest

The authors declare that they have no known competing financial interests or personal relationships that could have appeared to influence the work reported in this paper.
