# Supplementary material for: Differential early subcortical involvement in genetic FTD within the GENFI cohort
Source: Neuroimage Clin. 2021 Mar 29;30:102646. doi: 10.1016/j.nicl.2021.102646 (PMC8099608; doi:10.1016/j.nicl.2021.102646)

**Supplementary material**

**Supplementary Table 1:** **Volumetric comparisons for the brain regions between the different subgroups and the controls.** Volumetric comparisons, expressed as % of total intracranial volume, are adjusted for age, sex, total intracranial volume, and scanner type. Bold and italics represents a significant difference between groups after correcting for multiple comparisons. The % difference represents the volumetric difference between each group and controls. Abbreviations: Cortical: OF orbitofrontal, DLPFC dorsolateral prefrontal, VMPFC ventromedial prefrontal, Mot motor, AI anterior insular, PI posterior insular, TP temporal pole, DLT dorsolateral temporal, MT medial temporal, AC anterior cingulate, PC posterior cingulate, S sensory, MP medial parietal, LP lateral parietal, Occ occipital; Cerebellum: DN dentate nucleus, IN interposed nucleus, FN fastigial nucleus; Basal ganglia and Basal forebrain: NA nucleus accumbens, Cau caudate, Put putamen, GP pallidum, BF basal forebrain; Thalamus: AV anteroventral, LD laterodorsal, LP lateral posterior, VA ventral anterior, VLa ventral lateral anterior, VLp ventral lateral posterior, VPL ventral posterolateral, VM ventromedial, Int intralaminar, Mid midline, MD mediodorsal, LGN lateral geniculate nucleus, MGN medial geniculate nucleus, Pul Pulvinar; Amygdala: Sup superficial nuclei, CAT cortico-amygdaloid transition area, AB accessory basal nucleus; BL basal and paralaminar nuclei, LN lateral nucleus; Hippocampus: CA cornu ammonis, DG dentate gyrus, Sub Subiculum, Pre presubiculum; Brainstem: SCP superior cerebellar peduncle, MB midbrain, ME medulla; Hypothalamus: as anterior superior, ai anterior inferior, s-tub superior tuberal, i-tub inferior tuberal, pos posterior.

**Supplementary Figure 1. Plots representing the means and standard error bars for the whole brain volumes for each of the stages in *C9orf72*, *MAPT* and *GRN* mutation carriers.** Volumes as expressed as % the mean volumes in controls. * indicates a significant difference from controls after correcting for multiple comparisons.


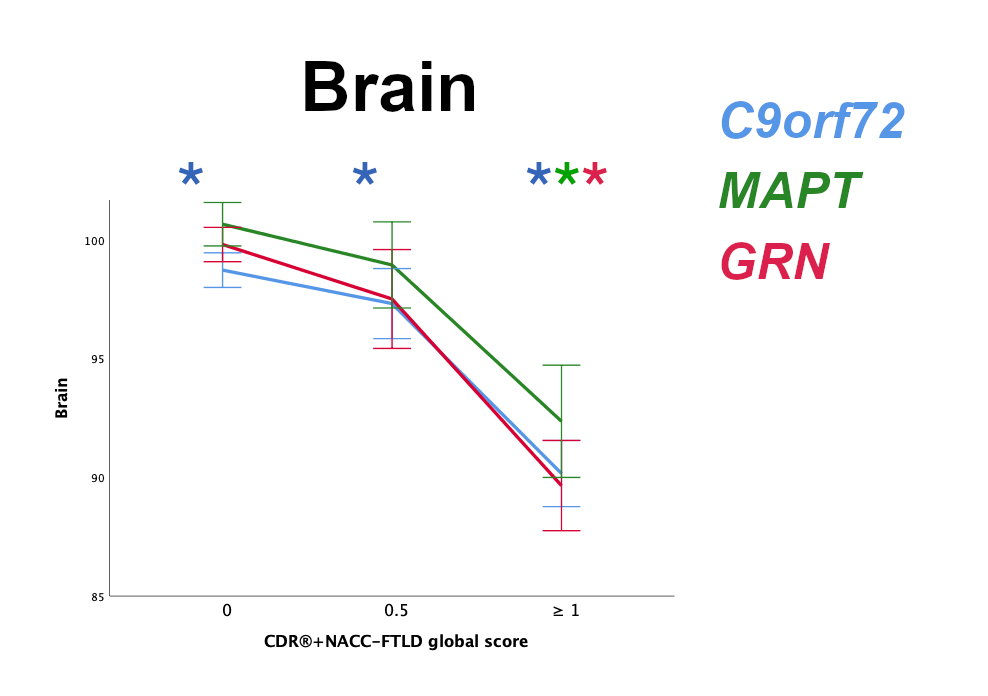


**Supplementary Figure 2. Plots representing the means and standard error bars for the cortical regions for each of the stages in *C9orf72*, *MAPT* and *GRN* mutation carriers.** Volumes as expressed as % the mean volumes in controls. * indicates a significant difference from controls after correcting for multiple comparisons.


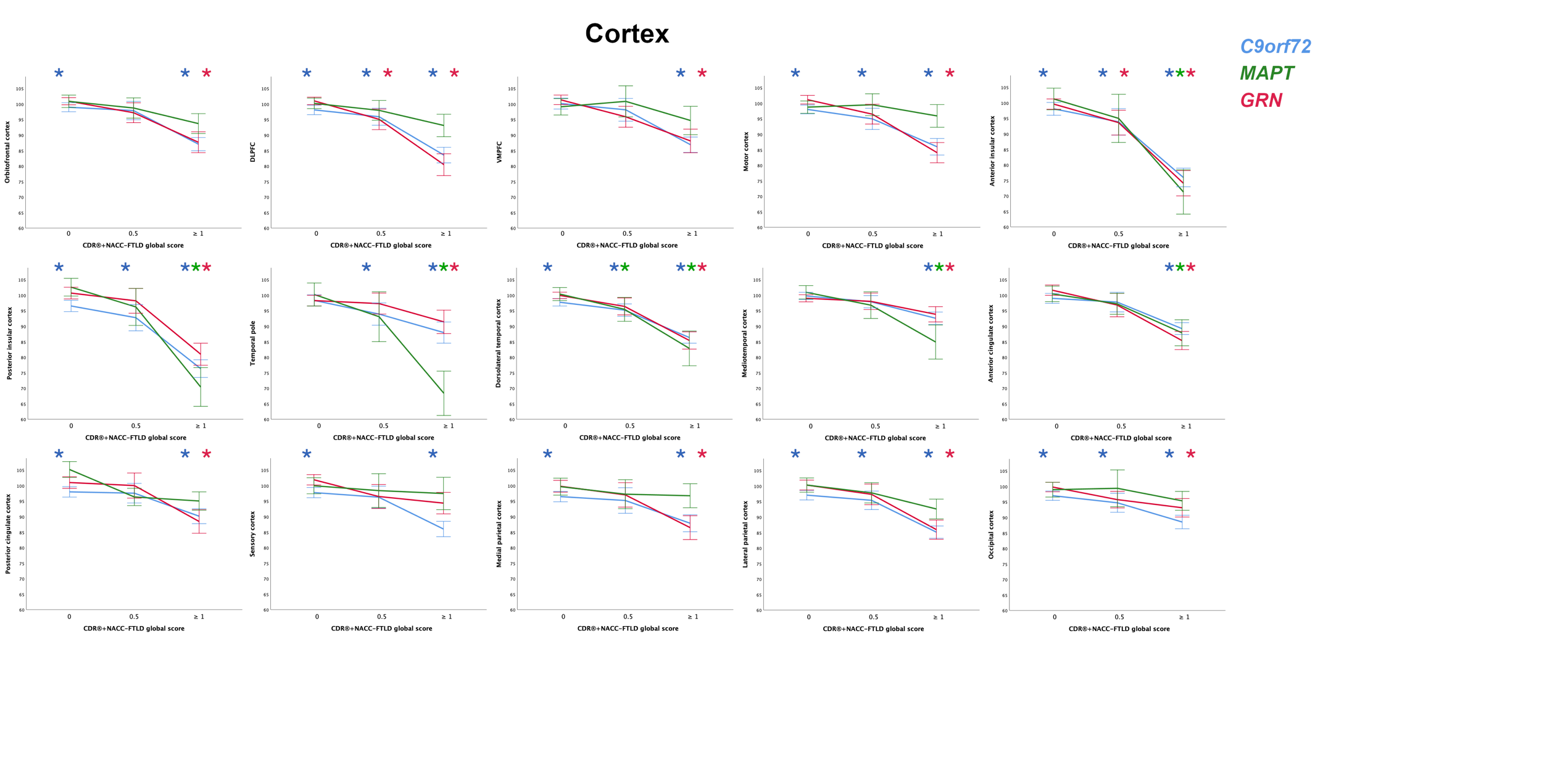

Supplement: Supplementary data 1 [file mmc1.docx]
